# Joint approach based on clinical and imaging features to distinguish non-neoplastic from neoplastic pituitary stalk lesions

**DOI:** 10.1371/journal.pone.0187989

**Published:** 2017-11-15

**Authors:** Ji Ye Lee, Ji Eun Park, Woo Hyun Shim, Seung Chai Jung, Choong Gon Choi, Sang Joon Kim, Jeong Hoon Kim, Ho Sung Kim

**Affiliations:** 1 Department of Radiology, Soonchunhyang University Bucheon Hospital, 170 Jomaru-ro, Wonmi-gu, Bucheon, Korea; 2 Department of Radiology and Research Institute of Radiology, University of Ulsan College of Medicine, Asan Medical Center, Seoul, Korea; 3 Department of Neurosurgery, University of Ulsan College of Medicine, Asan Medical Center, Seoul, Korea; Chinese Academy of Sciences, CHINA

## Abstract

**Purpose:**

Distinguishing non-neoplastic pituitary stalk lesions (non-NPSLs) from neoplastic pituitary stalk lesions (NPSLs) is a major concern in guiding treatment for a thickened pituitary stalk. Our study aimed to aid provide preoperative diagnostic assistance by combining clinical and magnetic resonance imaging (MRI) findings to distinguish non-NPSLs from NPSLs.

**Materials and methods:**

We recruited 158 patients with thickened pituitary stalk lesions visible on MRI. Laboratory findings included hypopituitarism, diabetes insipidus (DI), and hyperprolactinemia. MR images were assessed for anterior–posterior thickness (mm), diffuse pituitary stalk thickening, cystic changes, a high T1 signal, and glandular or extrasellar involvement. A diagnostic model was developed using a recursive partitioning logistic regression analysis. The model was validated in an independent dataset comprising 63 patients, and its diagnostic performance was compared with that of the original radiological reports.

**Results:**

A univariate analysis found significant associations of DI (*P* = 0.006), absence of extrasellar involvement (*P* = 0.002), and lower stalk thickness (*P* = 0.031) with non-NPSLs. A diagnostic model was created using the following parameters (in order of priority): 1) lack of extrasellar involvement, 2) stalk thickness < 5.3 mm, and 3) presence of DI. The diagnostic performance (area under the curve; AUC) of this model in the independent set was 0.813, representing a significant improvement over the original radiological reports (AUC: 0.713, *P* = 0.029).

**Conclusion:**

The joint diagnostic approach based on clinical and imaging-based factors robustly distinguished non-NPSLs from NPSLs. This approach could guide treatment strategies and prevent unnecessary surgery in patients with non-NPSL.

## Introduction

High-resolution magnetic resonance imaging (MRI; ≤ 3 mm) which allows the evaluation of features on both unenhanced and contrast-enhanced T1-weighted images, is currently the procedure of choice for evaluating sellar lesions [1]. A normal pituitary stalk has an anterior–posterior (AP) diameter of 3.25 ± 0.43 mm and a transverse diameter of 3.35 ± 0.44 mm at the level of the optic chiasm [2]. A broad range of diseases, including neoplasms, inflammatory disorders, and infection, cause thickening of the pituitary stalk [3]. However, the distinct criteria for a differential diagnosis remain uncertain. Several radiological studies have indicated that high-resolution MRI measurements of volume, symmetry, signal intensity, and stalk size increase the probability of a diagnosis of autoimmune hypophysitis [4]. Similarly, high-resolution MRI measurements of the fluid-fluid level, septation, location, and intracystic nodules can be used to distinguish Rathke’s cleft cysts from pituitary adenomas [5]. However, most previous studies focused on the pituitary gland, and no significant predictors specific for pituitary stalk lesions have been characterized.

The pituitary stalk plays a discrete functional role in the neural pathways of vasopressin and oxytocin [6]. Furthermore, pituitary portal vessels within the stalk transport various stimulatory and inhibitory factors of all pituitary hormones [6, 7]. Accordingly, patients with pituitary stalk lesions commonly present with varying degrees of diabetes insipidus (DI), hypopituitarism, and hyperprolactinemia [6]. However, metastatic tumors of the pituitary gland, especially the posterior lobe which contains a rich arterial blood supply may mimic pituitary stalk lesions but rarely give rise to clinical symptoms [8]. Thus, clinical findings may be useful in the differential diagnosis of pituitary stalk lesions.

Regarding the broad category of pituitary stalk lesions, the distinction of non-neoplastic pituitary stalk lesions (non-NPSLs) from neoplastic pituitary stalk lesions (NPSLs) is a major concern in terms of differential diagnosis. Neoplastic lesions usually require surgical resection, whereas non-neoplastic lesions can be followed up and successfully treated medically (e.g., corticosteroids for hypophysitis) [9, 10]. In addition, the unnecessary surgical excision of a non-NPSL can lead to serious complications, such as hypothalamic injury, iatrogenic pan-hypopituitarism, cerebrospinal fluid leakage, or infection [11, 12]. We hypothesized that a combination of clinical and imaging features might improve the diagnosis of pituitary stalk lesions, compared with MRI features alone. To date, most studies of the pituitary stalk have involved small patient cohorts and descriptive analyses [3, 13, 14]; in contrast, few studies have aimed to develop a diagnostic model by combining clinical and MRI findings. The purpose of our study, therefore was to distinguish non-NPSLs from NPSLs using a combination of clinical and MRI features.

## Materials and methods

### Patient data

The Institutional Review Board of Asan Medical Center approved this study ([http://eirb.amc.seoul.kr]:S2016-0555) and waived the requirement for written informed consent. We retrospectively reviewed the preoperative brain or sellar MRI scans and electronic medical records of patients between January 2009 and March 2016. A text search of the radiology reports during the study period, using the terms “pituitary stalk” and “infundibulum” and identified 2112 patients. Patients were subsequently excluded if 1) their radiological report read “normal pituitary stalk” and “normal pituitary infundibulum” (n = 1852), 2) they had a history of surgery or treatment adjacent to the pituitary stalk (n = 102). For the remaining 158 patients, the initial brain or sellar-specific MRI that identified a pituitary stalk abnormality was used to characterize the lesion. Laboratory, pathological, and clinical evaluations, and follow-up MRI analyses, were performed on clinically indicated cases. Patients were excluded if they required an endocrinological evaluation for the final diagnosis, but did not receive an endocrinological laboratory test.

The validation group was selected chronologically, and comprised 63 consecutive patients diagnosed with pituitary stalk lesions between April 2016 and September 2017. The reference standard for diagnosis was identical to that used for the training set and comprised a pathological or clinico-radiological diagnosis.

### Hormonal evaluation

Each patient’s hormonal status was evaluated before any medical treatment, surgical biopsy, or excision. The hormonal tests outlined below were performed for patients suspected to have anterior hypopituitarism. When the baseline serum measurements were abnormal, further dynamic confirmatory tests were performed. The gonadotropin axis was evaluated using the baseline serum levels of follicle-stimulating hormone (FSH), luteinizing hormone (LH), estradiol, and testosterone. The thyrotropin axis was evaluated by using the baseline serum levels of thyroid-stimulating hormone (TSH), free thyroxine, and free triiodothyronine. The growth hormone (GH) axis was evaluated using the serum levels of GH and insulin-like growth factor-1. The corticotropin axis was evaluated by baseline measurement and dynamic endocrine tests of serum cortisol, adenocorticotropic hormone (ACTH) at 8 am, an insulin-induced hypoglycemia test or a corticotropin test. Hypopituitarism was defined as more than one axis of deficiency of secretion of anterior pituitary hormones including FSH, LH, ACTH, TSH, and GH. Secondary hormonal deficiencies were diagnosed on the basis of low levels of primary hormones, with corresponding low levels of trophic pituitary hormones. Hyperprolactinemia was defined as a serum prolactin level exceeding 20 μg/L in patients without a history of risperidone or metoclopramide medication [15]. DI was diagnosed on the basis of typical signs and symptoms [16]; and documented through the measurement of sodium levels in the serum and urine as well as osmolality. More specifically, patients with DI have dilute urine (< 300 mOsm/kg H_2_O) with a urinary volume > 40 mL/kg/day. Twenty-one patients were subjected to a water deprivation test to further differentiate between partial central DI, partial nephrogenic DI, and primary polydipsia.

### Image acquisition

MRI was primarily performed using either a 3.0-T MRI scanner (Achieva or Ingenia; Philips Medical Systems, The Netherlands *or* Skyra; Siemens Healthcare, Germany) or a 1.5-T MRI scanner (Achieva; Philips), with an eight-channel head coil. The brain MRI protocol comprised a spin-echo sequence that included sagittal and axial T1-weighted imaging, axial T2-weighted imaging, axial fluid-attenuated inversion recovery imaging, and axial contrast-enhanced T1-weighted imaging. Following the injection of gadolinium based contrast (gadoterate meglumine), a gradient-echo, contrast-enhanced, T1-weighted image was obtained and reconstructed into the axial, coronal, and sagittal planes. The imaging parameters were as follows: 1) sagittal spin-echo T1-weighted images—repetition time (TR)/echo time (TE) = 450 msec/9.5 msec, section thickness = 5.0 mm, field of view (FOV) = 20 cm; 2) gradient-echo contrast enhanced T1-weighted images: TR/TE = 1800 msec/3.2 msec, section thickness = 3.0 mm, FOV = 25 cm. All patients underwent scanning with the enhanced brain MRI protocol.

In 78 patients who underwent sellar-specific MRI, high-resolution coronal T1-weighted imaging and T2-weighted imaging (section thickness: 2 mm, matrix: 512 × 512, FOV: 18 × 18 cm) were also available according to the following protocol: six coronal T1-weighted dynamic images were obtained every 25 seconds after an intravenous bolus injection (0.2 mL/kg) of contrast medium, and both coronal T1-weighted, contrast-enhanced imaging and sagittal T1-weighted imaging were performed. The imaging parameters for sagittal T1-weighted images were as follows: TR/TE = 450 msec/9.5 msec, section thickness = 2.0 mm, and FOV = 20 cm.

### Image analysis

**Fig 1:**
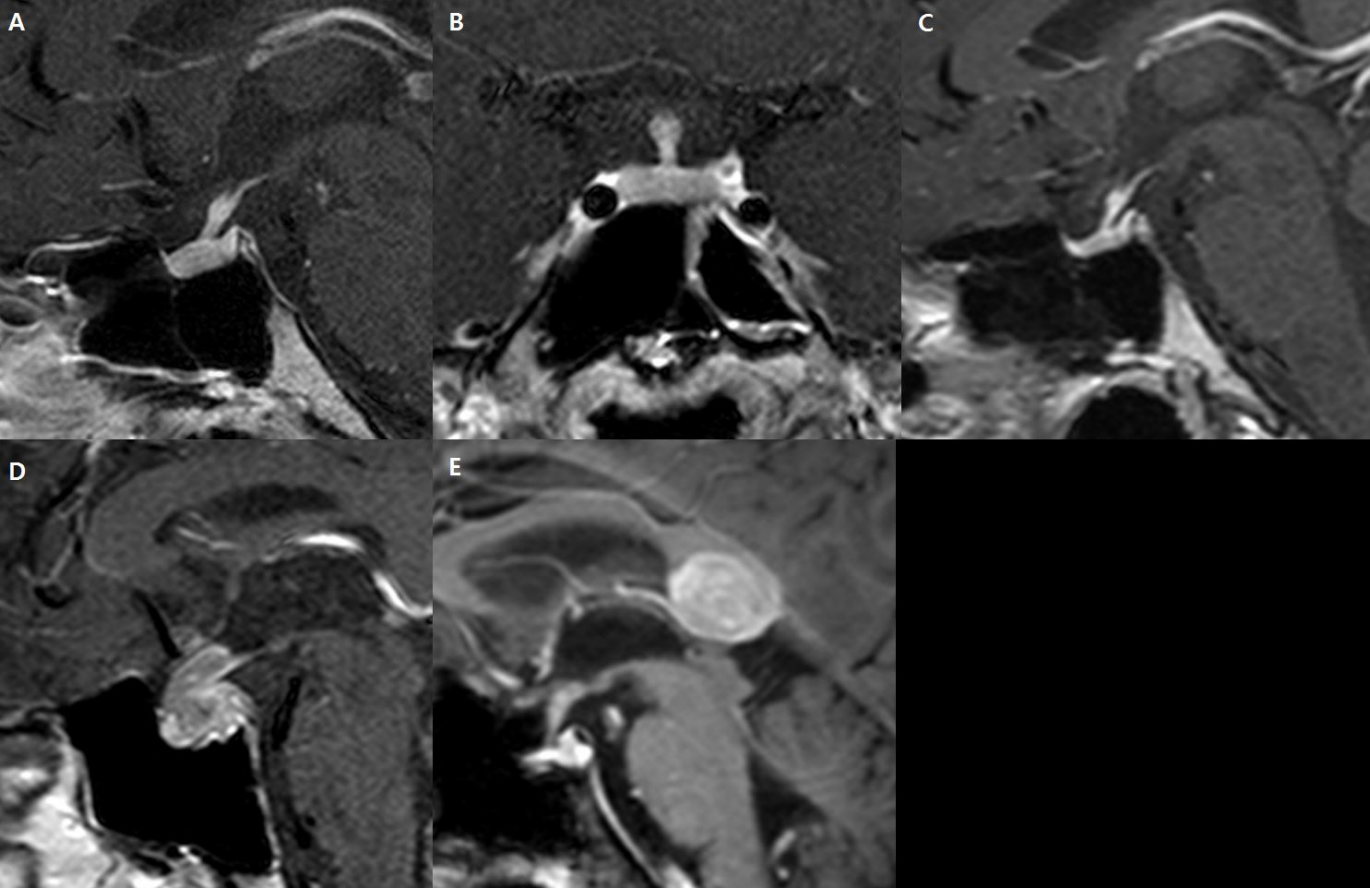
Typical magnetic resonance features of image analysis. (A) diffuse stalk thickening; (B) cystic change; (C) high T1 signal; (D) gland involvement; (E) extrasellar involvement.

Two independent neuroradiologists (J.E.P with 5 years and J.Y.L with 3 years of experience in neuroradiology) analyzed the MR images and recorded the following findings: AP thickness (mm), presence of a diffusely thickened pituitary stalk, cystic change, high T1 signal, pituitary gland involvement, or extrasellar involvement **(Fig 1)**.

The AP thickness was measured at the maximal diameter on a midline, sagittal, contrast-enhanced T1-weighted image. Diffuse stalk thickening was defined as a lesion with diffuse, uniform thickening without a nodular or fusiform shape. Cystic changes were evaluated using coronal or sagittal T2-weighted and contrast-enhanced T1-weighted images if a non-enhancing T2 high-signal area was present. High-T1 signal foci were identified on coronal or sagittal pre-contrast T1-weighted images if high signal foci relative to normal gray matter were detected. Extrasellar involvement was defined as the presence of a parenchymal or leptomeningeal enhanced lesion in other areas of the brain that were non-contiguous with the pituitary stalk or sellar fossa. Interobserver agreement was assessed, and discordant interpretations were resolved by consensus to create the diagnostic model. To determine AP thickness more precisely, the measured thicknesses obtained by the two readers were averaged.

### Reference standards for diagnosis

The reference standard for diagnosis was constructed in consensus between a neurosurgeon (J.H.K. with 22 years of experience in neurosurgery) and a neuroradiologist (H.S.K. with 15 years of experience in neuro-oncological imaging). All available laboratory, pathological, and clinical evaluations and all follow-up MRI images were reviewed. A pathological or clinico-radiological diagnosis was considered as reference standard. Among the included patients, 102 had a confirmed pathological diagnosis, and 56 had been diagnosed using clinic-radiological information. Fifty-six patients for whom pathological specimens were not available were diagnosed using clinico-radiological information when (1) tissue obtained from other areas of the brain and the lesion involving the stalk had similar imaging appearances and (2) tissue obtained from an extracranial lesion and follow-up MRI, clinical, and laboratory findings strongly suggested a specific diagnosis. We additionally attempted to reduce bias by including patients who were followed up for at least 1 year (median follow-up of 35.5 months). The neuro-radiologists who performed imaging analyses were blind to clinical information, which thus separated possible predictors and reference standards.

For instance, hypophysitis was diagnosed when the lesion decreased with corticosteroid treatment and no growth appeared during follow-ups. Metastasis was diagnosed when (1) a rapidly growing stalk lesion was detected, (2) surgery or biopsy from other brain areas or an extracranial site showed a primary malignancy, and (3) the imaging findings were similar in the pituitary stalk.

### Statistical analysis

The frequencies of imaging features of non-NPSLs and NPSLs were compared using the ***χ***^**2**^ test (for categorical variables) and t-test (for continuous variables). Interobserver agreement for each imaging feature was calculated using κ statistics.

The contribution of each imaging feature was evaluated using univariate and multivariate logistic regression models after differentiating non-NPSLs from NPSLs using a stepwise procedure. Based on these logistic regression analyses, a recursive partitioning tree classification algorithm was used to suggest a diagnostic tree model. An additional subgroup analysis of NPSLs was performed to identify significant predictors of pituitary metastases.

An independent radiologist used the validation set to make imaging diagnoses based on the suggested diagnostic model. The discriminatory powers of the diagnostic tree model were assessed using a receiver operating characteristic curve analysis, wherein the areas under the receiver operating characteristic curves (AUCs) were evaluated and compared using a conventional radiological report. To evaluate the influence of our combined approach, the AUCs of each imaging and clinical feature were compared with the results of the combined approach. A *P*-value of <0.05 was considered statistically significant. Statistical analyses were performed using the software package R, version 3.3.2 (http://www.R-project.org) and MedCalc Statistical Software version 17.1 (MedCalc Software, Ostend, Belgium).

## Results

### Comparison of clinical and imaging features between NPSLs and non-NPSLs

**Table 1**summarizes the diagnostic assignment of the pituitary stalk lesions.

**Table 1:** Final diagnoses of pituitary stalk lesions.

Classification	Number	Percentage (%)	External validation
**Means of establishing final diagnosis**			
Surgical/ pathological findings	102	64.6	41
Clinico-radiological diagnosis (follow-up≥ 1 year)	56	35.4	22
**Final diagnosis**			
**Non-neoplastic disease**			
Lymphocytic hypophysitis	21	13.3	18
Granulomatous hypophysitis	1	0.6	1
IgG4 related hypophysitis	1	0.6	0
Xanthogranulomatous disseminatum	1	0.6	0
Tuberculosis	2	1.3	0
Cholesterol granuloma	1	0.6	0
Neuromyelitis optica	1	0.6	0
Sarcoidosis	0	0	1
**Neoplasm**			
Schwannoma	1	0.6	0
Meningioma	1	0.6	0
Pituitary carcinoma	1	0.6	0
Germ cell tumor	26	16.5	7
Langerhans cell histiocytosis	4	2.5	1
Craniopharyngioma	23	14.6	4
Choroid plexus carcinoma	1	0.6	2
Pleomorphic xanthoastrocytoma	1	0.6	2
ALL/lymphoma	18	11.4	5
Pilocytic astrocytoma	6	3.8	1
Anaplastic astrocytoma	1	0.6	0
Glioblastoma	1	0.6	1
Metastasis	46	29.1	20
**Total**	158	100	63

Note- primary malignancies in metastasis: lung cancer (n = 27), breast cancer (n = 11), stomach cancer (n = 5), skin cancer (n = 1), melanoma (n = 1), and thyroid cancer (n = 1).

Among non-NPSLs, lymphocytic hypophysitis was the most frequent diagnosis (13.3%), followed by tuberculosis (1.3%). In NPSLs, metastasis was the most frequent diagnosis (29.1%), followed by germ cell tumor (16.5%), craniopharyngioma (14.6%), and hematological malignancy (11.4%).

**Table 2**summarizes the clinical and imaging features of the patients with pituitary stalk lesions.

**Table 2:** Comparison of clinical and imaging features between non-neoplastic and neoplastic pituitary stalk lesions.

Variables	Non-neoplastic lesions (n = 28)	Neoplastic lesions (n = 130)	*P-*value
Age, years	48.1 ± 17.8	48.4 ± 17.1	0.938
Sex, male/female ratio	13:15	75:55	0.010 ^a^
MR field strength (1.5: 3.0T)	9:19	34:96	0.06 ^a^
Clinical features			
Diabetes insipidus	19 (76)	59 (44.4)	0.006 ^a^
Hypopituitarism	17 (68)	68 (51.1)	0.493 ^a^
Hyperprolactinemia	11 (44)	46 (34.6)	0.419 ^a^
Imaging features			
AP thickness (mm)	5.3 ± 3.7	10.1 ± 8.1	0.004
Diffuse stalk thickening	12 (48)	22 (16.5)	0.057 ^a^
Cystic change	6 (24)	33 (24.8)	0.937 ^a^
T1 high signal	4 (16)	23 (17.3)	0.392 ^a^
Gland involvement	13 (52)	73 (54.9)	0.569 ^a^
Extrasellar involvement	1 (4)	66 (49.6)	**0.005** ^a^

Results are shown as means ± standard deviations for continuous variables and numbers with percentages (in parentheses) for categorical variables

^a^ From the ***χ***^**2**^ test

Twenty-eight and 130 patients had non-NPSLs and NPSLs, respectively. The groups were not imbalanced in terms of age (mean ± standard deviation: 48.1 ± 17.8 vs. 48.4 ± 17.1 years, *P =* 0.938) and magnetic field strength for MR imaging (1.5: 3.0 T, 9:19 vs 34:96, *P* = 0.06). The male-to-female ratio was higher in the neoplastic group than in the non-neoplastic group (percentage of men: 46.4% in the non-neoplastic group vs. 57.7% in the neoplastic group, *P* = 0.01). Patients with non-NPSLs had a significantly higher rate of DI (76%, *P* = 0.006), compared to those with NPSLs.

The interobserver agreements for imaging features were excellent with κ values of 0.879 for the presence of a diffusely thickened pituitary stalk, 1.0 for cystic changes, 0.98 for a T1 high signal, 0.98 for pituitary gland involvement, and 0.99 for extrasellar brain involvement.

Among the imaging features, the AP thickness on sagittal images was significantly larger in patients with NPSLs (mean ± standard deviation, 10.1 ± 8.1 mm) than in those with non-NPSLs (5.27 ± 3.69 mm, *P* = 0.004). Patients with non-NPSLs more frequently had diffuse stalk thickening, but this was not a significant finding. In contrast, extrasellar involvement was observed more often in patients with NPSLs (*P* = 0.005).

Among a subgroup analysis of NPSLs, a multivariate analysis identified an older age and extrasellar brain involvement as significant predictors of pituitary metastases. The result in the NPSL group is shown in **Table 3**.

**Table 3:** Clinical and imaging predictors for pituitary metastases among neoplastic pituitary stalk lesions.

	Univariate analysis		Multivariate analysis	
Variables	Odds ratio (95% CI)	*P* Value	Odds ratio (95% CI)	*P* Value
Clinical features				
Age	1.07 (1.04–1.10)	<0.001	1.06 (1.03–1.10)	<0.001
Sex	1.69 (0.82–3.52)	0.156		
Diabetes insipidus	0.24 (0.11–0.54)	<0.001		
Hypopituitarism	2.13 (1.02–4.46)	0.04		
Hyperprolactinemia	0.19 (0.07–0.50)	<0.001		
Imaging features				
AP thickness	0.91 (0.86–0.97)	0.001		
Diffuse stalk thickening	1.19 (0.46–3.09)	0.72		
Cystic change	0.21 (0.07–0.63)	0.002		
T1 high signal	0.51 (0.17–1.47)	0.19		
Gland involvement	0.58 (0.28–1.23)	0.15		
Extrasellar involvement	7.28 (3.10–17.07)	<0.001	7.75 (2.42–24.8)	<0.001

Note: CI; confidence interval, AP; anterior to posterior

### Developing a diagnostic model

In the univariate logistic regression analysis of clinical and imaging features, the presence of DI (odds ratio [OR]: 3.97, *P* = 0.006), a lower AP thickness value (OR: 0.83, *P* = 0.011), the presence of a diffusely thickened pituitary stalk (OR: 4.66, *P* < 0.001), and the lack of extrasellar brain involvement (OR: 0.04, *P* = 0.002) were identified as potential predictors of non-NPSLs **(Table 4).**

**Table 4:** Clinical and imaging predictors of non-neoplastic and neoplastic stalk lesions.

	Univariate analysis		Multivariate analysis*	
Variables	Odds ratio (95% CI)	*P* Value	Odds ratio (95% CI)	*P* value
Clinical features				
Age	2.13 (0.89–5.08)	0.977		
Sex	2.13 (0.89–5.08)	0.089		
Diabetes insipidus	3.97 (1.49–10.58)	0.006	3.75 (1.14–12.39)	0.030
Hypopituitarism	2.03 (0.82–5.03)	0.126		
Hyperprolactinemia	1.49 (0.62–3.54)	0.370		
Imaging features				
AP thickness	0.83 (0.72–0.96)	0.011	0.88 (0.78–0.99)	0.031
Diffuse stalk thickening	4.66 (1.88–11.55)	<0.001	3.42 (0.99–11.81)	0.052
Cystic change	0.96 (0.35–2.60)	0.931		
T1 high signal	0.91(0.28–2.91)	0.875		
Gland involvement	1.12 (0.48–2.64)	0.790		
Extrasellar involvement	0.04 (0.01–0.32)	0.002	0.04 (0.01–0.32)	0.002

Note- CI; confidence interval, AP; anterior to posterior

*Multivariate analysis after stepwise regression

In the multivariate stepwise regression, all above-listed variables remained independent factors that could distinguish non-NPSLs from NPSLs.

**Fig 2:**
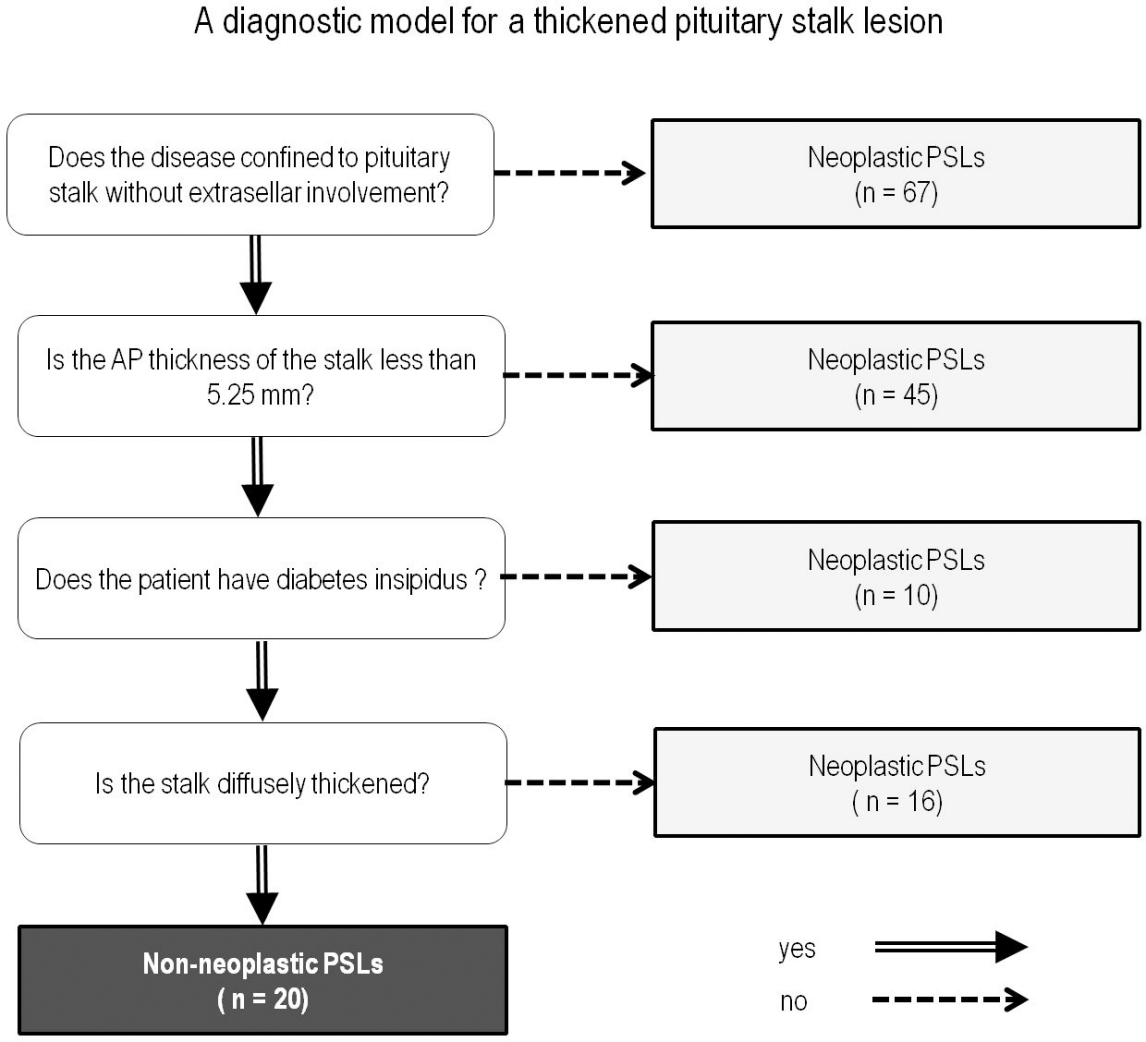
A diagnostic model for a thickened pituitary stalk lesion in distinguishing non-neoplastic pituitary stalk lesions from neoplastic pituitary stalk lesions in the training set, based on recursive partitioning analysis. PSLs, pituitary stalk lesions.

Next, a recursive decision tree was created using the above mentioned significant variables in the training set data (S1 File). Four terminal nodes were produced in five splits. These terminal nodes were as follows (in order of priority): presence of extrasellar involvement, AP thickness with a cutoff of 5.25 mm, presence of DI, and presence of a diffusely thickened pituitary stalk. The established diagnostic model is shown in **Fig 2**.

**Fig 3:**
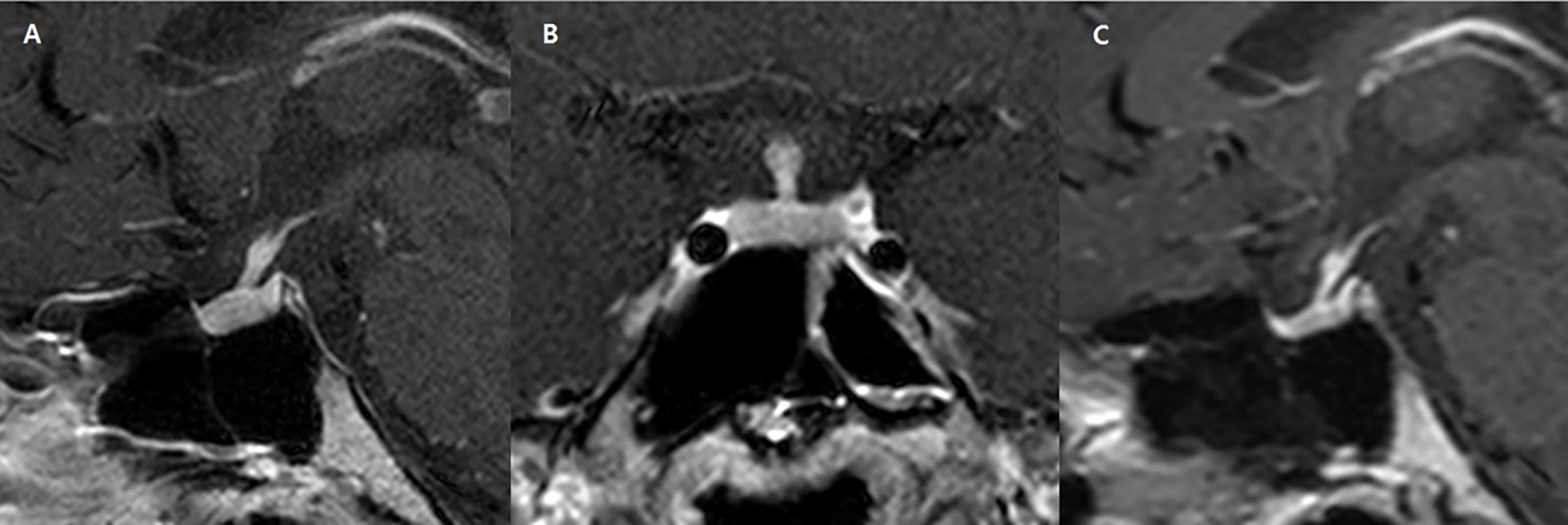
A 25-year-old woman with pan-hypopituitarism and pituitary stalk thickening (maximal anterior–posterior diameter: 2 mm). (A) The patient presented with clinical features of diabetes insipidus and a diffuse pattern of stalk thickening was observed. (B) Similar finding on coronal image, and this lesion was classified as a non-neoplastic based on the diagnostic model, and was diagnosed as hypophysitis via clinico-radiological follow-up after steroid therapy. (C) After 1 year of follow-up, the infundibular thickening had improved.

**Fig 4:**
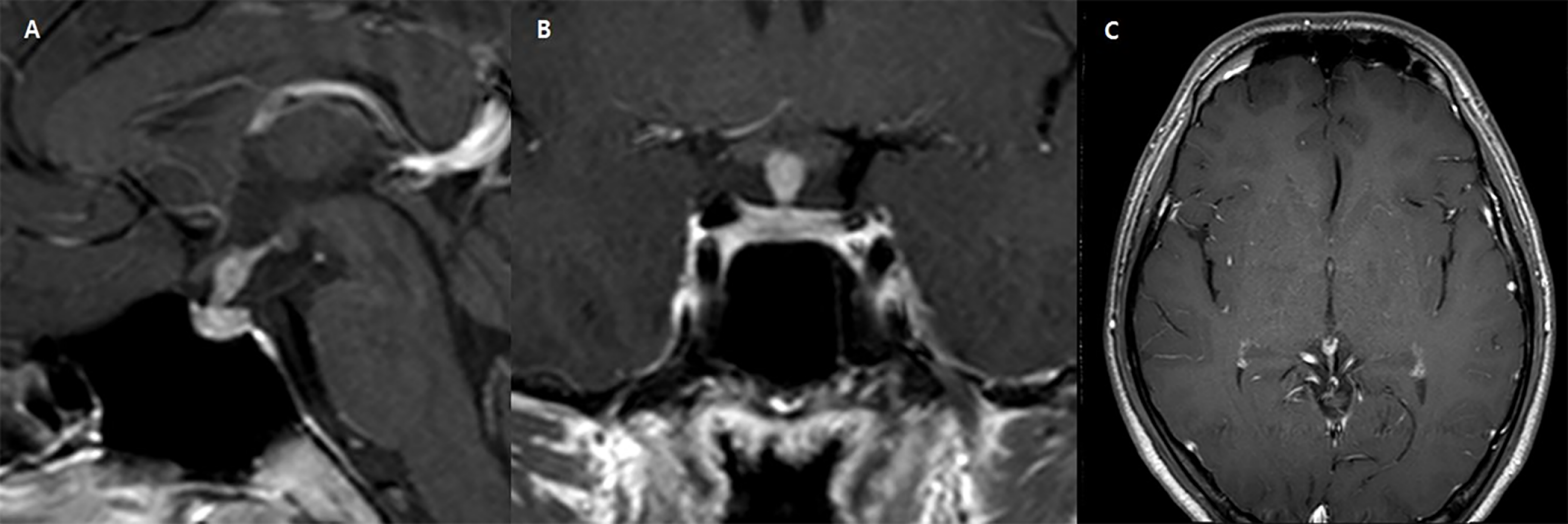
A 21-year-old man with pituitary stalk thickening. (A) The patient presented with symptoms and laboratory findings consistent with diabetes insipidus, showing thickened pituitary stalk with a maximal anterior-posteriro diameter of 5mm. (B) The stalk showed fusiform and non-diffuse thickening. (C) There was no remarkable abnormality in the pineal gland. The lesion was classified as a neoplastic pituitary stalk lesion based on the diagnostic model, and finally diagnosed as a germinoma via biopsy.

The diagnostic model correctly classified 141 (89.2%) of the 158 cases in the training set. Regarding non-NPSLs, the sensitivity and specificity of the diagnostic model were 65.5% and 94.6%, respectively. A receiver operating characteristic curve analysis was performed to compare the AUC of the diagnostic tree model with that of the radiological report. The AUC value of the diagnostic model was 0.828 (95% confidence interval [CI] 0.759–0.883), compared with 0.70 (95% CI 0.623–0.771) for the radiological reports; this difference was statistically significant (*P* = 0.037). **Figs 3 and 4**show representative cases of non-NPSL and NPSL.

### Validation and performance of the diagnostic model

The proposed diagnostic model was validated in an independent data set of 63 cases (20 non-NPSLs and 43 NPSLs). No significant differences were found between the study and validation groups in terms of age, sex, or final diagnosis.

**Fig 5:**
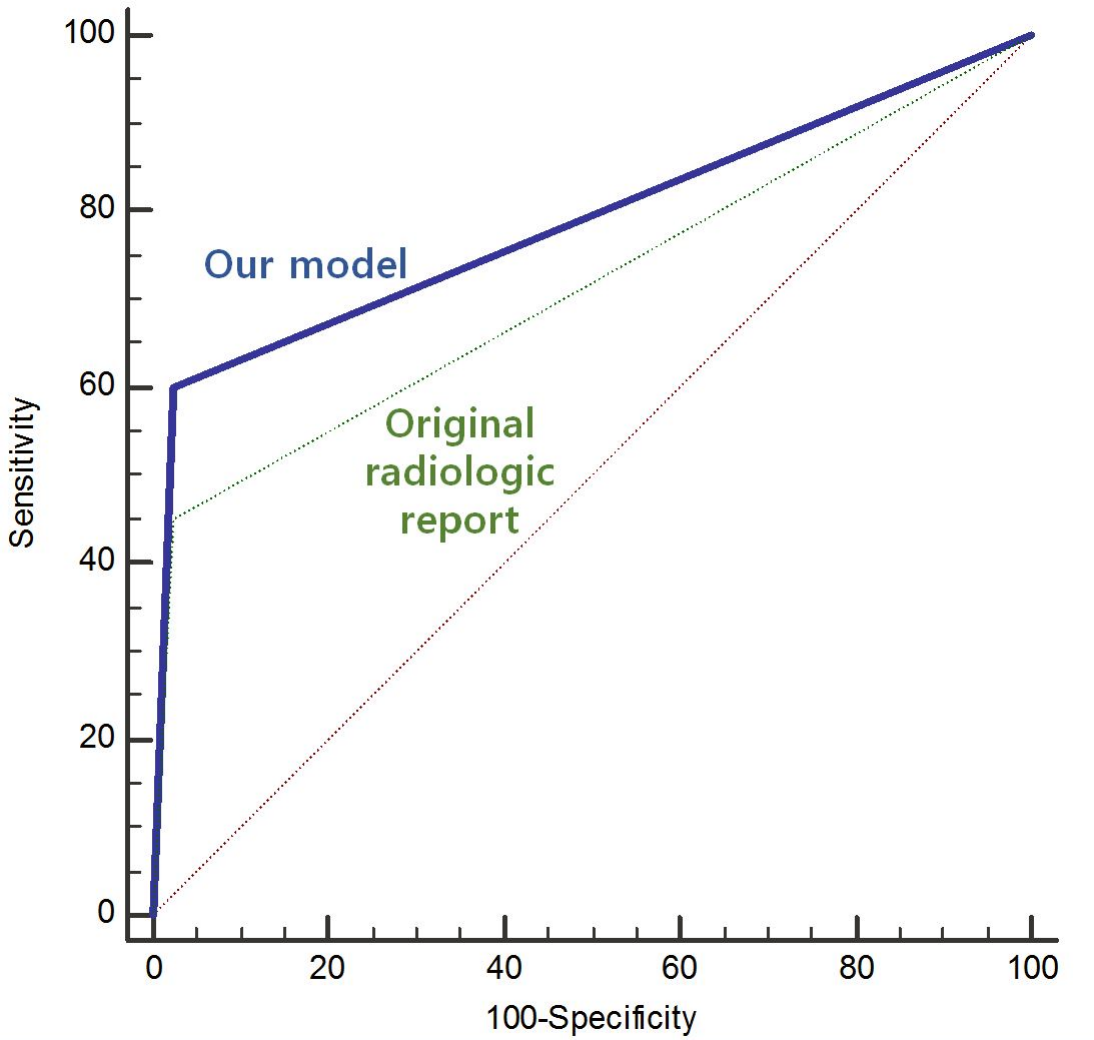
Receiver operating characteristic curve comparison of our diagnostic model with the original radiological report in terms of differentiating non-neoplastic from neoplastic pituitary stalk lesions in the validation set.

Fifty-five of these 63 cases (87.3%) were classified correctly. The diagnostic performance of the model in the validation set was 0.813 (AUC, 95% CI [0.695–0.900]), which was a significant improvement over the original radiological report (AUC: 0.713, 95% CI [0.586–0.820], *P* = 0.029, **Fig 5**). This joint approach yielded the highest diagnostic performance when compared with the clinical feature of DI (AUC, 0.747, 95% CI [0.622–0.848]) or the following individual imaging features: extra-sellar involvement (AUC, 0.787, 95% CI [0.666–0.880]), size (AUC, 0.751, 95% CI [0.626–0.851]), or diffuse stalk thickening (AUC, 0.695, 95% CI [0.567–0.805]).

## Discussion

The present study suggests that a diagnostic approach incorporating clinical and MRI features of thickened pituitary stalk lesions could increase the probability of distinguishing non-NPSLs from NPSLs. This distinction is important, because non-NPSLs such as autoimmune hypophysitis can be treated medically, whereas NPSLs ultimately require surgery [3, 17]. Our diagnostic approach yielded diagnostic performances of 0.83 (AUC) in the training set and 0.81 in the validation set. Furthermore, our approach represented a significant improvement over the original radiological reports. Given its simplicity and convenience, this joint approach may be useful for preoperative diagnosis in patients with a thickened pituitary stalk.

Among the various imaging features of the pituitary stalk, only diffuse thickening was found to significantly associate with non-NPSLs, whereas neither cystic changes nor high T1 signal was a significant predictor. Diffuse stalk thickening may reflect the pathological features of lymphocytic hypophysitis, such as the diffuse infiltration of mainly mature lymphocytes, with islets of fibrosis and histiocytes [17, 18]. Indeed, this condition was predominant among cases of non-NPSL in our population. On the other hand, an intrinsically high T1 signal in the pituitary posterior lobe, which reflects functional vasopressin storage [19], is frequently reported to be lost in cases of autoimmune hypophysitis but is conserved in the majority of pituitary adenomas [4]. However, many descriptive studies concerning the differentiation of pituitary stalk lesions did not report a relationship between a T1 high signal and the enhancement pattern [3, 13, 14]. Our findings support the scenario, wherein a loss of high T1 signal intensity does not help to differentially diagnose thickened pituitary stalk lesions, as both non-NPSLs and NPSLs involve the pituitary stalk itself.

Conversely, the imaging features indicative of NPSL were distinct, and included a thickened pituitary stalk with a diameter exceeding 5.25 mm and extrasellar brain involvement. The difference in stalk thickness between non-NPSLs and NPSLs may be attributable to a difference at the time of clinical presentation, as studies have shown that the mean onset of clinical presentation of a pituitary tumor is 23 ± 35 months [20], whereas that of hypophysitis is 10 ± 18 months [21]. Regarding extrasellar involvement, non-NPSLs may exhibit a mass-like configuration and mimic sellar or suprasellar tumors [4, 22] but are have less likely to aggressively invade the surrounding suprasellar and bony structures. In addition, non-NPSLs may also be associated with extrasellar brain parenchymal lesions, such as neurosarcoidosis or tuberculosis [23, 24], although these entities are relatively uncommon among non-NPSLs. The only patient with extrasellar brain involvement in the present study had tuberculous meningitis, with leptomeningeal enhancement in the basal cistern.

Interestingly, the most useful laboratory predictor of non-NPSLs was the presence of DI. This can also be a clinical manifestation of NPSLs, as the posterior lobe has a rich arterial blood supply and can therefore be directly involved in metastasis through the systemic circulation [25]. In our study, the significant predictor of DI may correlated closely with the onset of clinical presentation. Previous studies have shown that lymphocytic or granulomatous infiltration can induce early, direct, and definitive damage to pituitary cells [17, 26], whereas in cases of stalk metastasis, tumor cell infiltration is associated with a delayed onset of DI [8], particularly when extrinsic compression predominates over destructive changes. This finding is also supported by our data that 21.7% of patients with metastases (10/46 patients) and 76% of patients with non-NPSLs had DI. More importantly, our results demonstrated an improvement in diagnostic performance when using laboratory findings vs. imaging features such as the presence or loss of a T1 bright high signal, which indicates a loss of posterior pituitary function. Conversely, the anterior pituitary function, or hypopituitarism/ hyperprolactinemia, was not a useful parameter for differentiating non-NPSLs from NPSLs.

Transsphenoidal surgery is a safe and effective treatment for sellar lesions [4, 27]. However, the pituitary stalk plays a critical role in hormonal function, and unnecessary surgery must be avoided. Previous research has revealed that imaging features differ from clinical features in a descriptive sense. Nonetheless, more analytical methods, such as measuring the importance of certain features, may guide clinicians when making differential diagnoses, especially when distinguishing non-NPSLs from NPSLs. Our study which included a large data set, identified an AP stalk thickness threshold of 5.25 mm using routinely performed, sagittal T1-weighted images with a slice thicknesses ranging from 3 to 5 mm. These MRI parameters could be easily applied in daily clinical practice. Furthermore, the diagnostic performance was improved by combining both clinical and imaging features, in contrast with the original radiological reports from the present study. Moreover, our study found robust results for the validation set.

However, our study was subject to several limitations. First, the retrospective nature of the study introduced the risk of selection bias. Our exclusion of patients with insufficient follow-up, as well as those who lacked a pathological specimen, may have excluded many patients with non-NPSLs resulting in a larger number of patients in the neoplastic group. Second, hormonal tests were indicated clinically, and not all patients underwent testing for the entire pituitary axes of the anterior and posterior pituitary glands. Further studies involving with complete laboratory tests of the entire pituitary axes might help to identify possible predictors among hormonal axes. Third, neither a quantitative analysis nor lesion signal enhancement was possible given the heterogeneous nature of the MRI machines used (1.5 T and 3.0 T). We attempted to minimize the effects of different magnetic field strengths by comparing the lesion signal with that of normal grey matter. In addition, our analysis did not use advanced MRI techniques, including dynamic contrast-enhancement (DCE)-MRI of the sellar fossa. A recent study [28] reported that DCE-MRI helps to localize and characterize pituitary lesions; therefore, a future study should use the homogenous pulse sequence to characterize the signal intensity and dynamic pattern of contrast enhancement. Fourth, although our diagnostic model was tested on an independent data set, the patients were recruited from the same institution; therefore, the study is somewhat limited in terms of generalizability. A multicenter study with a large number of patients may further support our results. Finally, MRI characteristics are routinely implemented into clinical contexts, and the clinical and radiological predictors suggested in this study are not a completely new concept. However, our study is valuable because it has attempted to set an order of priority for the use of various clinical and MRI characteristics when for distinguishing non-NPSLs from NPSLs.

In conclusion, our study identified that the absence of extrasellar brain involvement, diffuse stalk thickening < 5.25 mm, and the presence of DI as significant predictors that differentiate non-NPSLs from NPSLs. Our proposed diagnostic model combined clinical and MRI characteristics to improve the diagnostic performance relative to the original radiological report. Such a model would facilitate decisions regarding further treatment strategies in clinical settings.

## Supporting information

S1 File The clinical and imaging features of the pituitary stalk lesions in the training set. Abbreviations: tumor_dx, tumor diagnosis (1: neoplastic, 0: non-neoplastic); dx_1, neoplastic lesion coded as 1; masslike, mass-like configuration; T1high, T1 high signal intensity; h_side, hypothalamic side; p_side, pituitary side; offtarget, presence of extrasellar lesion; DI, diabetes insipidus; hyperpRL, hyperprolactinemia; size (mm).
(CSV)
